# UV-shielding and wavelength conversion by centric diatom nanopatterned frustules

**DOI:** 10.1038/s41598-018-34651-w

**Published:** 2018-11-02

**Authors:** Edoardo De Tommasi, Roberta Congestri, Principia Dardano, Anna Chiara De Luca, Stefano Managò, Ilaria Rea, Mario De Stefano

**Affiliations:** 1 National Research Council, Institute for Microelectronics and Microsystems, Unit of Naples, Naples, I-80131 Italy; 2 University of Rome “Tor Vergata”, Department of Biology, Laboratory of Biology of Algae (LBA), Rome, I-00133 Italy; 3 National Research Council, Institute of Protein Biochemistry, Naples, I-80131 Italy; 4 University of Campania “Luigi Vanvitelli”, Department of Environmental, Biological and Pharmaceutical Sciences and Technologies, Caserta, I-81100 Italy

## Abstract

Diatoms can represent the major component of phytoplankton and contribute massively to global primary production in the oceans. Over tens of millions of years they developed an intricate porous silica shell, the frustule, which ensures mechanical protection, sorting of nutrients from harmful agents, and optimization of light harvesting. Several groups of microalgae evolved different strategies of protection towards ultraviolet radiation (UVR), which is harmful for all living organisms mainly through the formation of dimeric photoproducts between adjacent pyrimidines in DNA. Even in presence of low concentrations of UV-absorbing compounds, several diatoms exhibit significant UVR tolerance. We here investigated the mechanisms involved in UVR screening by diatom silica investments focusing on single frustules of a planktonic centric diatom, *Coscinodiscus wailesii*, analyzing absorption by the silica matrix, diffraction by frustule ultrastructure and also UV conversion into photosynthetically active radiation exerted by nanostructured silica photoluminescence. We identified the defects and organic residuals incorporated in frustule silica matrix which mainly contribute to absorption; simulated and measured the spatial distribution of UVR transmitted by a single valve, finding that it is confined far away from the diatom valve itself; furthermore, we showed how UV-to-blue radiation conversion (which is particularly significant for photosynthetic productivity) is more efficient than other emission transitions in the visible spectral range.

## Introduction

Detrimental effects of ultraviolet radiation (UVR) exposure on living organisms, especially UVC (100–280 nm) and UVB (280–315 nm), are well known. Among them, the predominant lesion on DNA is the formation of dimeric photoproducts between adjacent pyrimidines^1^. These lesions result in structural changes in DNA molecules which interfere with RNA transcription and DNA synthesis, causing misreading or erroneous replication of the genetic code and leading to mutation or cell death^2^.

The natural source of UVR *par excellence* is the Sun. Radiation characterized by wavelengths <200 nm is absorbed by atmospheric nitrogen and oxygen, while the remaining fractions of UVC and UVB are efficiently filtered by stratospheric ozone, with an absorption peak centered at 250 nm. A small amount of unabsorbed UVB still reaches the Earth surface and ecologically significant depths in coastal and oceanic waters^2,3^, causing direct DNA damages and being injurious for both phototrophs and heterotrophs^3^. Studies conducted by means of ultraviolet submersible spectroradiometers^3^ allowed detection of UVB in ocean depths down to 60–70 m, thus threatening life in the euphotic zone.

As far as phytoplanktic microalgae are concerned, UVB effects include inhibition of cell division and of photosynthesis; changes to the main photosynthetic enzyme, ribulose bis-phosphate carboxylase; a decrease in nutrient uptake and in metabolism and protein synthesis; and loss of photo-orientation and motility^2,4^.

Several molecular mechanisms allow protection of phytoplankton cells from UVR. They comprise photoenzymatic repair (PER) photoreactivation, which consists of direct monomerization of cyclobutane dimers by photolyase in the presence of visible and UVA light^2,5^; synthesis of photoprotective pigments such as carotenoids (e.g. diadinoxanthin and beta-carotene)^3^, accumulated in the cytoplasm and acting as passive sunscreen^6^; and also that of mycosporyne-like amino acids (MAAs), which absorb between 268 and 362 nm^7^ based on their structure, with maximum absorption ranging between 309–362 nm^8^, excluding most of UVB. MAAs accumulate in response to high light exposure^3,4,6^ and also osmotic stress. Despite these photoprotection mechanisms, a great variability in UV susceptibility and consequent induced damages has been observed across microalgal genera and even species^2,3,9^, probably due to different cell morphologies, placement of organelles (possible chloroplast shielding or nuclear hiding), different concentrations of UV-absorbing pigments, and DNA content (e.g. genomes with high thymine content will lead to a considerable proportion of lesions due to thymine-thymine cyclobutane dimers)^2^. In particular, several diatoms, especially those that are radially symmetric (centrics) show high tolerance to UVB^2,3,10^ in natural populations, even if the overall community MAA content is found to be low^4,10^. In some cases the concentration of UV-absorbing compounds in diatom populations has been estimated to be 2 to 5 orders of magnitude lower per cell unit than in *Phaeocystis*^10^, a major component of Antartic phytoplankton communities.

Diatoms are ubiquitous, unicellular microalgae living in all oceans and freshwaters. They are responsible for about 20–25% of global primary production^11^ and derived oxygen. Their protoplasm is enclosed in a hydrated porous amorphous silica shell called *frustule*, which is formed by two valves interconnected by a lateral cingulum containing micrometric and submicrometric pores. According to frustule symmetry, diatoms can be distinguished in *centrics*, characterized by a radial symmetry of the valves, and *pennates*, which are bilaterally symmetric. Until now, about 10^5^ species have been estimated. In some cases, it has been observed that frustules act as mechanical barriers and selective filters against grazers and pathogens, respectively. This led to the hypothesis that these abilities may be shared by most species^12,13^. Furthermore, the similarity of these structures to artificial photonic crystals led to the study of their ability to manipulate light^14–19^. Potential exploitation of optical, mechanical and structural properties of diatom frustules has been predicted and tested in several fields: design and development of highly efficient solar cells^20–22^; plasmonics and Surface Enhanced Raman Spectroscopy (SERS)^23–25^; sub-diffractive optics^26^; photoluminescence-based sensors and biosensors^27,28^; molding in micro- and nano- devices fabrication^29^; nanovectoring of therapeutic agents in cancer cells^30,31^; random lasing and dye trapping^32,33^, with several further innovative applications repeatedly envisaged^34^.

Centric and pennate diatoms seem to have developed strategies for UVR protection that go beyond the synthesis of MAAs and UV absorbing pigments. Usually benthic diatoms, which are mostly bilateral in symmetry (pennates), inhabit environments less exposed to UV such as sediments and microbial mats^2^, and are motile, although non flagellated as all diatoms, thus escaping high light levels by migration downwards, as is the case with *Gyrosigma balticum*^35^. Planktic diatoms comprise mainly centric forms living suspended in the water column and they are still characterized by a noticeable UVR tolerance. Low induction of lesions after UV irradiation has been observed for *Thalassiosira australis* compared to other planktonic microalgae^2^; UVB inhibition of growth rate is much greater for *Phaeocystis* than for a clone of *Chaetoceros socialis*^3^, despite the fact that in this species no detectable MAAs have been observed by High Performance Liquid Cromatography (HPLC)^4^. Furthermore, survival of *Thalassiosira tumida* and *Stellarima microtias* under UVB illumination do not show any significant decline until very high irradiances^10^. A study conducted on phytoplankton tow and sediment samples from Southern Ocean showed that the presence of MAAs is tightly associated with the biosilica matrix of the frustules, but detectable only after severe treatment in hydrofluoric acid, thus suggesting that concentration and diversity of MAAs in diatoms may be higher than previously expected^36^. However, a comprehensive study on 152 species of microalgae^4,8^ grown in culture under white fluorescent light, showed low levels of UV-absorbing compounds in diatoms when compared to dinoflagellates, cryptophytes, pymnesiophytes and raphidophytes.

The first centric diatom fossils date back to Cretaceous (about 120 Myr ago)^37^, but further studies^12^ indicate that they originated close to the Permian-Triassic boundary, about 250 Myr ago. In those geological periods the oxygen content (and, consequently, the ozone content) of the atmosphere was considerably lower than today^38^, and the exposure to UVB and UVC radiation was higher. Thus it follows that the process of silification (and then the formation of the frustule) developed in a phase of transition between (relatively) low content of atmospheric oxygen and a geological period of higher oxygen content. This could lead to the conjecture that frustules allowed centric diatoms to adapt to a high UVR environment^10^, thus explaining their considerable tolerance towards UV irradiation. However, the exact mechanisms by which this tolerance takes place are still not clear: survival of diatoms does not necessarily correlate with absorption by either pigments, oxidisable cell contents or other known screening mechanisms^10^.

Prior results and numerical simulations suggest that single valves of two centrics, *Coscinodiscus wailesii* and *Arachnoidiscus* sp., collect and confine photosynthetically active radiation (PAR, *λ* = 400–700 nm) with high efficiency but do not show the same behavior for UVB radiation^16,39,40^. Attenuation of transmittance in UV has also been recently observed in more or less uniform monolayers and multilayers of dried frustules or rinsed cells^41–43^, but it is quite difficult to discriminate the possible origin of the effect from single frustule structure, quantity of material, and spectral properties of diatom layers.

In this paper we investigated possible mechanisms by which a single valve of *Coscinodiscus wailesii* Gran & Angst, whose optical properties have been deeply analyzed over the last ten years^15–18,44–46^, may be able to protect the cell from harmful radiation, considering both UVB and UVC radiation. In particular, we identified three main mechanisms contributing to cell protection: absorption by silica and trace organic compounds incorporated in the frustule; diffraction (due to the geometry of the valve and refractive index contrast respect to the surrounding environment); and photoluminescence (mainly due to chemical surface defects of hydrated amorphous porous silica). These processes have been studied by means of transmission measurements, Raman and photoluminescence spectroscopies, and numerical simulations based on Beam Propagation Method (BPM) corrected for wide angles.

## Results and Discussion

### *C. wailesii* frustule

**Figure 1:**
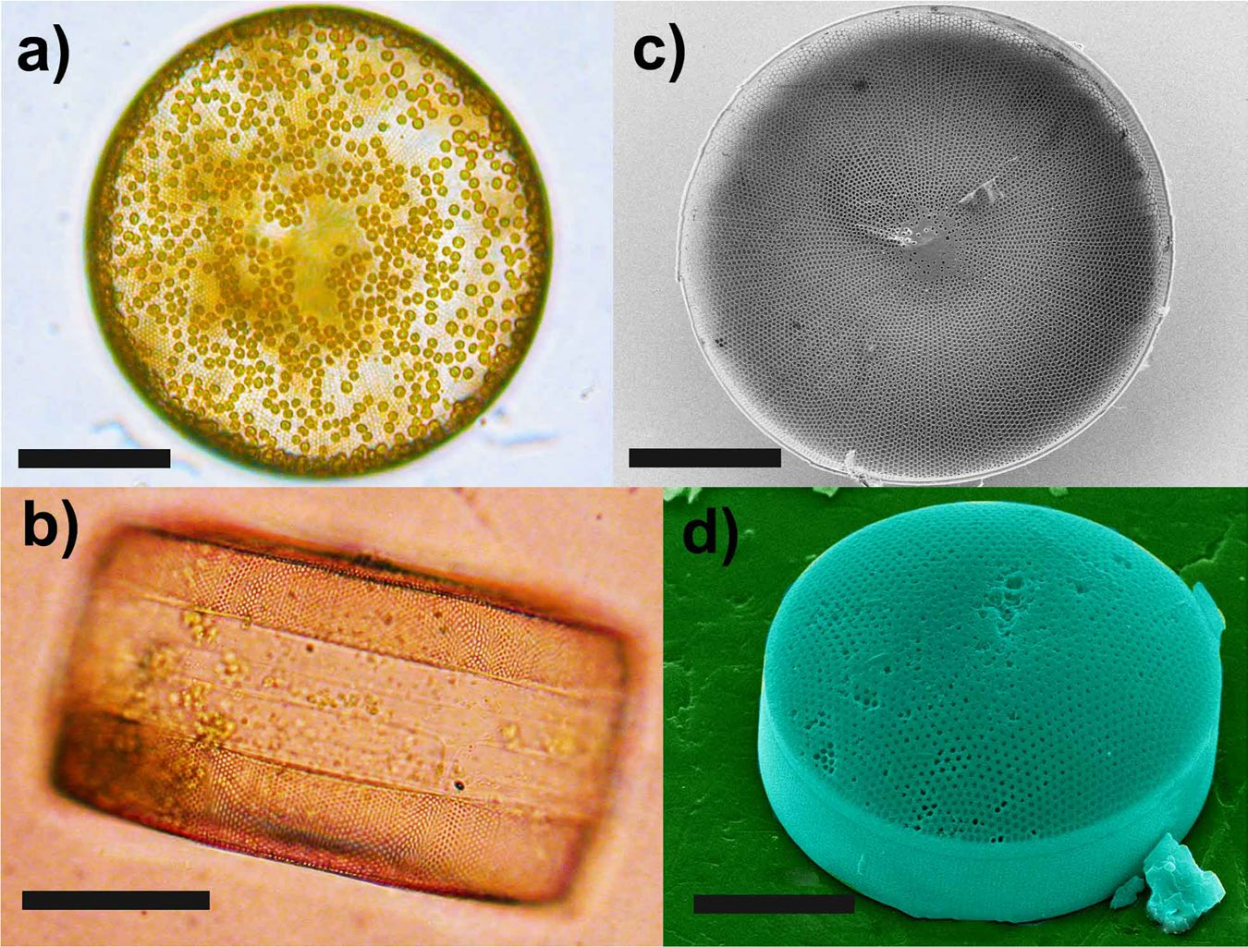
Light micrographs of a living cell in face view (**a**) and side view (**b**); many small discoid chloroplasts are visible. FESEM micrographs of a cleaned single valve, inner view (**c**) and outer view of a complete theca (**d**). Scale bars: 50 *μ*m.

Light microscope observations of monoalgal culture of the strain CCAP 1013/9 of the centric diatom species *C. wailesii* allowed for observation of cells that are circular in valve face (Fig. 1a) and barrel shaped in a side view, with a pervalvar axis smaller than a mean diameter of about 150 *μ*m, containing many small discoid chloroplasts. A side view showed the valve mantle forming a right angle with the valve face and visible margins of *cingulum* mainly composed of two bands (Fig. 1b). Field-Emission Scanning Electron Microscopy (FESEM) observations revealed that valves and girdle bands are characterized by quasi-periodic patterns of pores whose diameter and lattice constant depend on location. Radial rows of *areolae* are visible on the valve face, both the inner and side view, with a hyaline central area (Fig. 1c,d).

**Figure 2:**
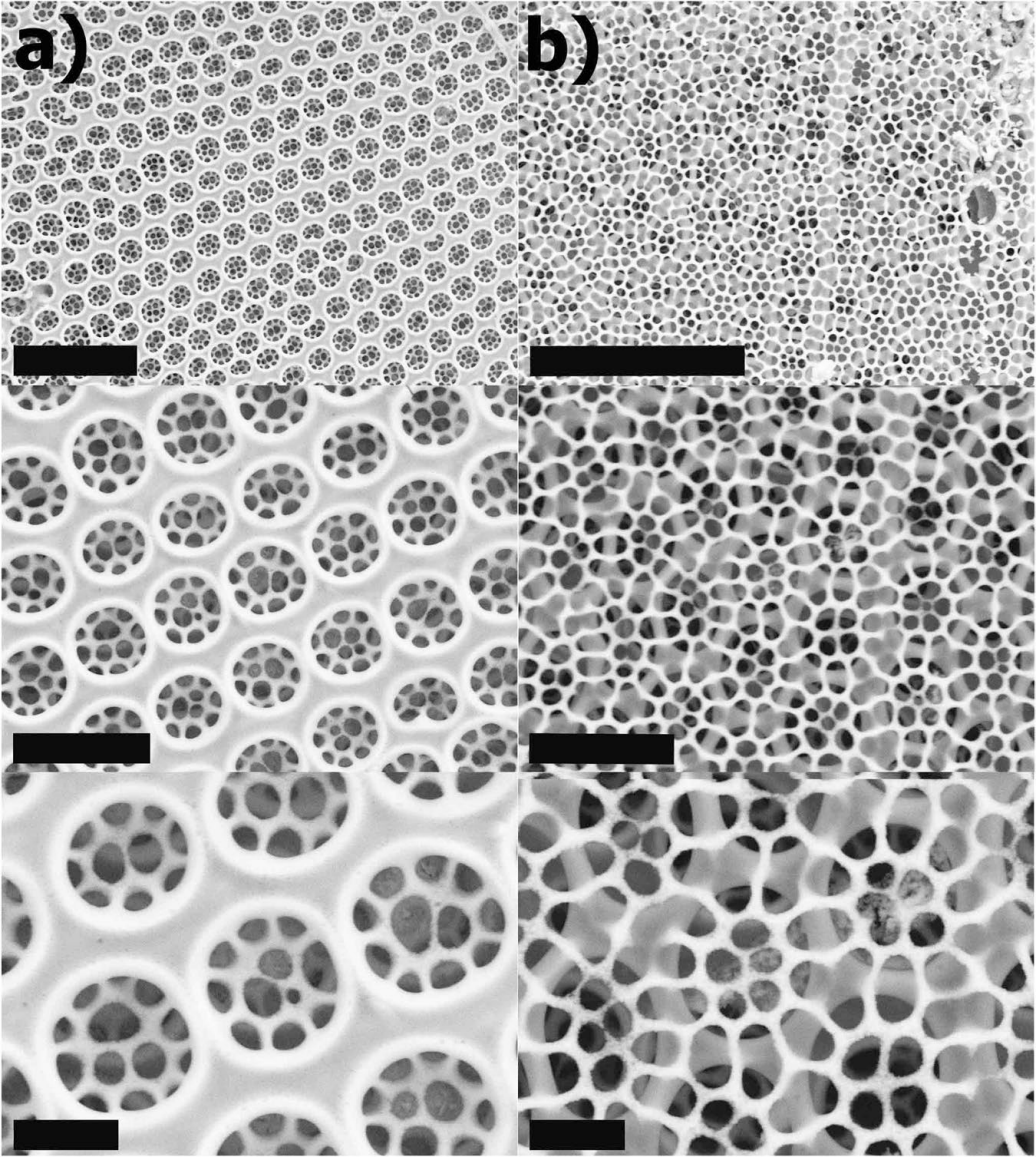
Details of the inner (**a**) and external (**b**) plate of a single valve of *C. wailesii* diatom obtained by FESEM with progressive higher magnification. Scale bars: 10 *μ*m (first row); 3 *μ*m (second row); 1 *μ*m (third row). The ultrastructure of the frustule, with pores of different dimensions, periodicity and lattice constant in different plates, is clearly visible. In particular, the ultrastructure of the outer plate is clearly visible through pores of the inner silica layer and vice versa.

Figure 2 shows that the inner valve plate presents pores of 1.4 ± 0.1 *μ*m in diameter (see Fig. 2a) arranged in an hexagonal pattern with a lattice constant of 1.8 ± 0.1 *μ*m; pores of the outer plate (Fig. 2b) have a more complex shape and arrangement, characterized by a mean equivalent diameter of 350 ± 100 nm and a lattice constant of 420 ± 50 nm.

### Transmission of optical radiation through a single valve

**Figure 3:**
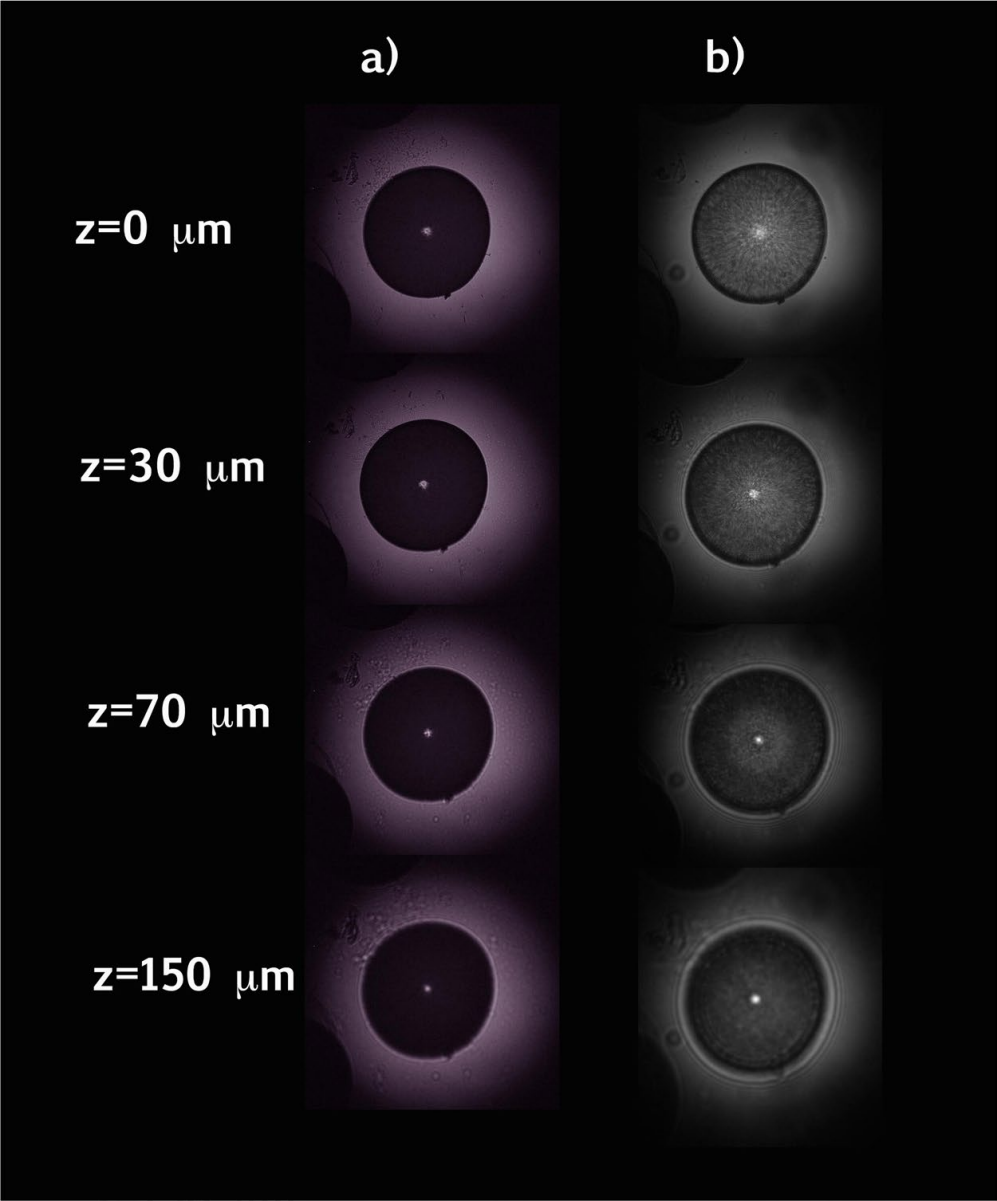
Microscope images of a single valve at different distances from the lying plane for UVC (**a**, *λ* = 250 nm) and visible-near IR radiation (**b**, *λ* = 400–1100 nm).

Diatoms probably originated in low ozone conditions. Thus, we investigated at first the interaction of a *C. wailesii* valve with UVC radiation at 250 nm (ozone absorption peak) and compared results with those relative to PAR and near IR interaction. In Fig. 3, left side, images of the valve invested by radiation emitted by a deuterium lamp (*λ* = 200–400 nm) and filtered at 250 ± 5 nm are shown at different distances from the valve itself. On the right side, the valve is irradiated by a tungsten alogen lamp (*λ* = 400–1100 nm).

**Figure 4:**
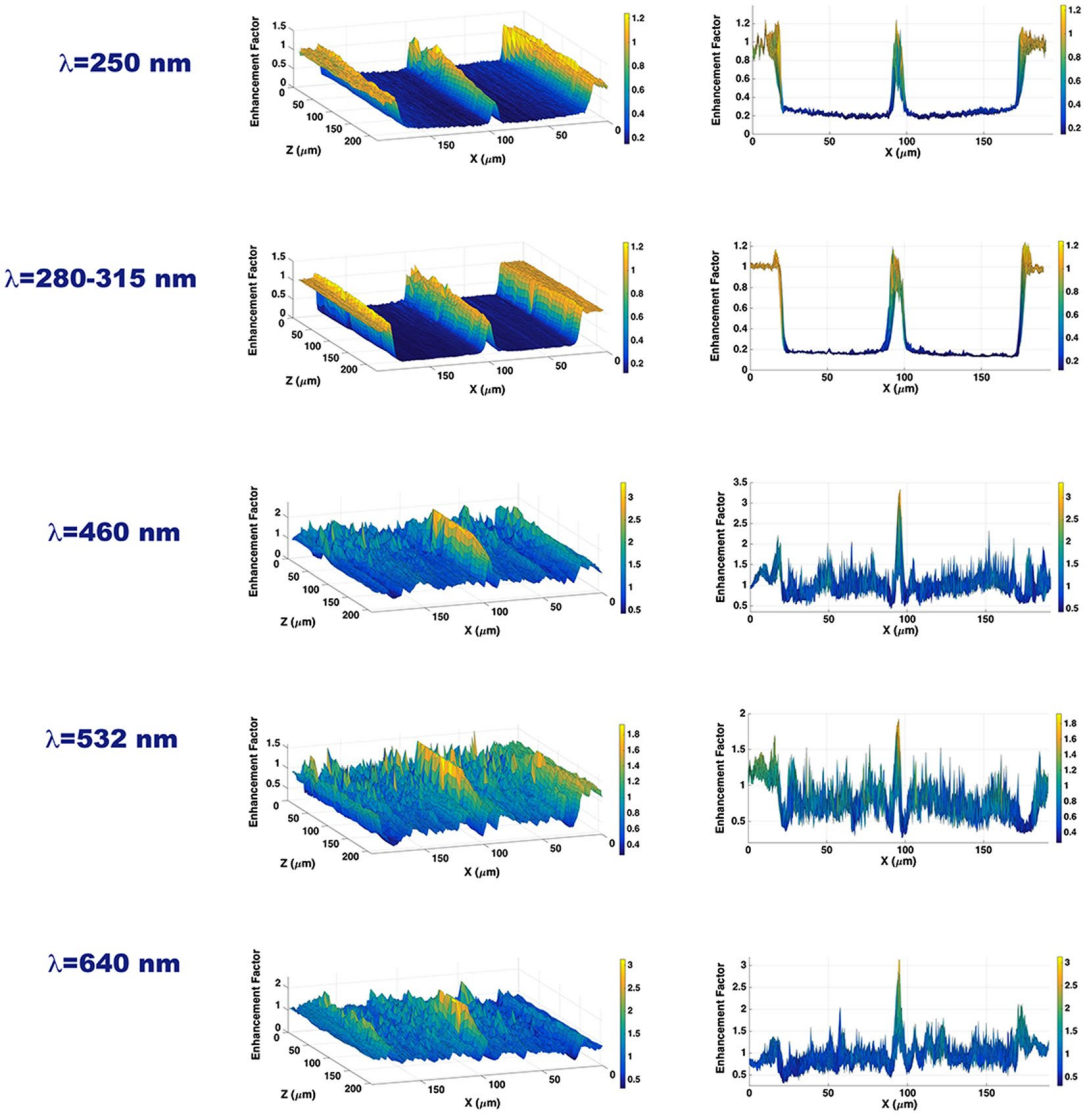
Enhancement Factor (defined as the transmitted to incident intensity ratio) along the optical axis for different wavelengths. On right column, frontal views of the profiles are reported.

The hydrated silica of the valve showed strong absorption of UVR while appearing generally more transparent to visible and near IR radiation. Furthermore, we observed a different spatial behavior of the light along the optical axis. In correspondence with specific distances and for visible-near IR radiation, light is confined in intense hot-spots. The origin of these spots, far from being due to refraction and lens-like effects, are ascribed to coherent superposition of diffractive contributions coming from the single pores, as widely demonstrated both numerically and experimentally^15–18,40,46,47^ and described in the following paragraphs. In case of UVC irradiation, the central spot did not seem to increase in intensity as a function of the distance from the valve. We evaluated the Enhancement Factor (EF), defined as the ratio between transmitted and incident intensity, measuring it along a diameter of the valve, then increasing the distance from the valve itself for different spectral intervals as shown in Fig. 4. It is convenient to express this quantity as EF and not as transmittance since for visible radiation it reaches values above 1, due to spatial redistribution of transmitted intensity caused by the confinement effect.

In the first two rows of Fig. 4 the behavior at 250 ± 5 nm and at UVB (radiation filtered between 280 and 315 nm) is analyzed. As can be seen, in both cases, the radiation is attenuated about 80% along the valve, while the central spot never significantly overcomes the incident radiation in intensity. Furthermore, in the case of UVC radiation the intensity of the central spot tends to attenuate along the optical axis. As we will see in next paragraphs, this is to be ascribed to a combination of absorbing and diffractive effects. In the following rows, the same analysis for blue (460 ± 5 nm), green (532 ± 5 nm) and red (640 ± 5 nm) radiation is reported. In these relatively narrow spectral ranges, PAR is absorbed by chlorophylls and carotenoids. In particular, the photosynthesis action spectrum (defined as the photosynthetic rate per unit of incident irradiance versus wavelength) is characterized, for most of microalgae, by two maxima in the blue and red ranges^48^. It is thus interesting to notice that, for blue and red radiation, the intensity in the central spot is amplified over three times the incident radiation. Due to the optical properties of hydrated amorphous silica^49^, in this case no significant absorption is detected if compared to UV irradiation, but still some light, away from the hotspot, is lost due to scattering and/or reflection.

### Absorption contribution

As outlined before, absorption is the most straightforward mechanism that could be involved in UV shielding by hydrated, porous, amorphous silica that constitutes diatom frustules^50^. For wavelengths below 200 nm, the strong absorption of silica is mainly due to the interaction of radiation with electrons of Si-O bonds and with point defects such as -OH groups (which, of course, are abundant in hydrated silica), Si-Si bonds and strained Si-O-Si bonds^51^. The higher the level of impurity, the higher the wavelength at which the so called “absorption edge” (i.e. the sharp cut-off in silica absorption spectrum) takes place. The absorption edge could be at about 160 nm (i.e. silica could be transparent above 160 nm and opaque below) in the case of a high level of purity, but the presence of inclusions and point defects shifts it towards visible wavelengths. On the contrary, for crystalline forms of silica such as quartz, the absorption edge moves toward short wavelengths^51^.

**Figure 5:**
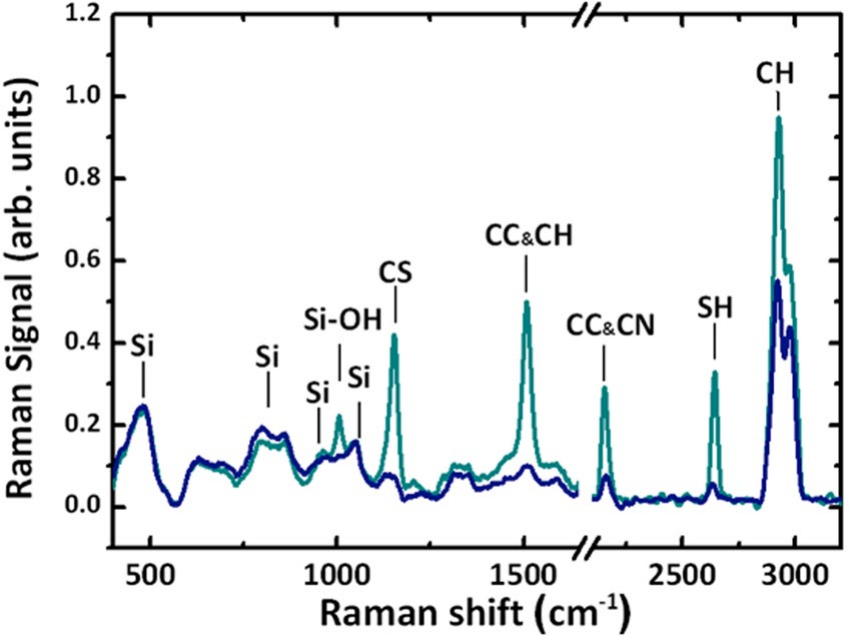
Raman spectra of a single valve of *C. wailesii* over the range 400–3200 cm^−1^. The spectra were acquired in different points of the valve and then mediated. Blue spectra: mean over 22 spectra. Cyano spectra: mean over 3 spectra where presence of sulfur composites was detected. Complete band assignments are reported in Table 1.

**Figure 6:**
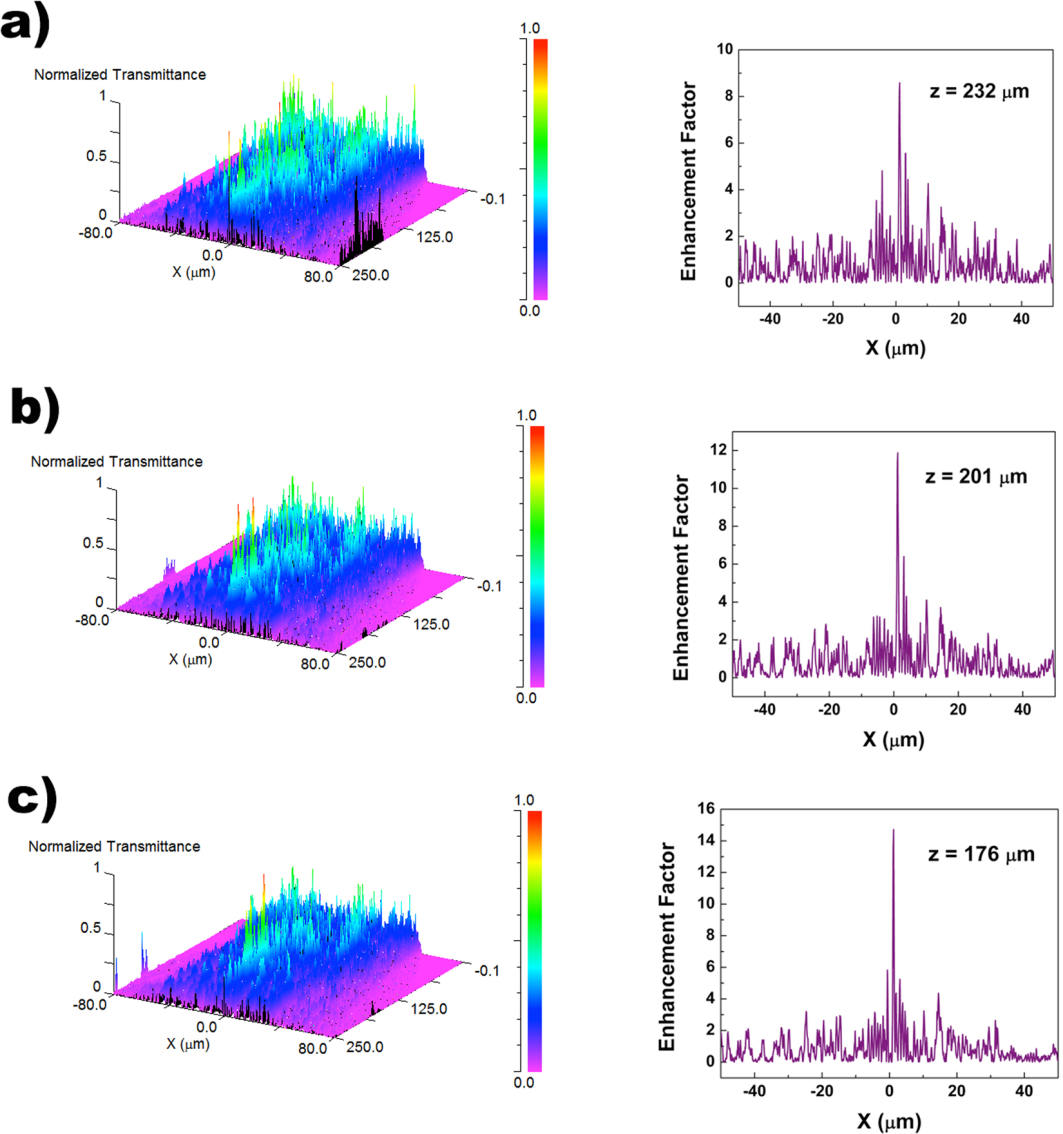
Intensity spatial distribution in *XZ* plane of UV radiation transmitted by a single valve in air (left column); intensity profile of the first hot-spot with indication of its position along the optical z axis (right column). The simulations have been performed for *λ* = 250 nm (**a**), 280 nm (**b**) and 315 nm (**c**).

The bulk composition of *C. wailesii* frustules and the possible presence of impurities which could contribute to UV absorption was screened here by Raman spectroscopy. Figure 5 shows mean Raman spectra acquired in different locations of a single *C. wailesii* valve. Apart from the expected spectral signatures proper of hydrated silica (i.e. SiO_2_, Si-O-H and Si-O-Si bonds), specific bands coming from C-C and C-H bonds have been detected. This is ascribed to residual organic compounds incorporated in the porous walls of the diatom^52^. Indeed, Kammer *et al*. already detected, by FT-IR and Raman imaging analysis, the presence of organic impurities in *Stephanopyxis turris* valves, even after treatment with acid solutions commonly used to remove the organic components and clean frustules. More interestingly, the spectra in Fig. 6 show additional Raman signals associated to C=S and S-H bonds. While the peaks relative to C-C, C-H, and silica bonds were present in all the acquired spectra, the peaks relative to sulfur bonds were found only in few valve locations and were characterized by a higher intensity, indicating that the spatial distribution of sulfur composites was not uniform and was more localized.

**Table 1:** Band assignments for the Raman spectra reported in Fig. 5.

Band position (cm^−1^)	Band assignments^52,71–73^
450–550	Si-O-Si stretching
800–850	SiO_4_ symmetric stretching
900–1050	SiO_2_, Si-OH stretching
1050–1100	SiO, SiO_2_, SiO_3_ symmetric stretching
1150–1200	C=S stretching
1450–1550	CH, CH_2_, CH_3_, C=C
2100–2250	C≡C, C≡N
2550–2600	SH stretching
28000–3000	CH, CH_2_, CH_3_ stretching

Centric diatoms and, in general, all phytoplankton organisms, are involved in the sulfur global cycle^53^. Indeed dimethyl sulfide (DMS) mostly originates from phytoplankton secondary metabolites and is then transferred to the atmosphere, representing the main natural source of atmospheric sulfur. Oxidized DMS is in turn involved in sulfur aerosols production and a relative change in the numbers of cloud condensation nuclei, affecting solar radiation scattering. The resulting variation in the radiative balance of the Earth affects phytoplankton growth and associated sulfur production; in this way the sulfur cycle is finally self-regulated^54^.

Sulfur compounds are also connected to frustule morphogenesis. The role of sulfhydryl groups in silicates uptake by diatoms was firstly experimentally observed in the Fifties by Lewin^55^. In 1998 Hildebrand and coworkers^56^ identified five silicon transporter genes in *Cylindrotheca fusiformis* whose encoded aminoacid sequences present nine conserved cysteines, which contain sulfur as part of their chemical structure. The Hildebrand studies paved the way to the discovery, analysis and classification of all the polypeptides and proteins involved in silica precipitation, biosilica synthesis and frustule formation (mainly silaffins, long-chain polyamines, frustulines and cingulines^13^).

### Diffraction contribution

Numerical simulations based on the Beam Propagation Method and corrected for wide angles (see Methods and references^16,40^ for details) allowed for the study of how the valve interacts with an incoming plane wave at different wavelengths. SEM images of the inner and outer surface of the *C. wailesii* valve have been converted into binary refractive-index maps. The maps have been properly extruded (allowing for simulation of three-dimensional light propagation) and positioned in order to obtain the bilayer geometry characteristic of the valve. The valve defines the *XY* plane. The incoming fields were launched along the *Z* axis and the valve extends from *z* = 0 *μ*m to *z* = 1 *μ*m. The output of the simulation is the spatial distribution of the transmitted intensity in *XZ* and *YZ* planes. The value of refractive index is, of course, a function of wavelength. The simulation did not take into account the imaginary part of the refractive index, so attenuation due to absorption from silica was not reproduced. This kind of analysis, thus, allowed discriminating the diffractive effects of the nanopatterned valve on incoming optical fields from the absorption contributions experimentally determined and reported above.

At first, UV irradiation in air has been analyzed. In Fig. 6, both the intensity distribution in the *XZ* plane (left column) and the position of the first hot-spot along the *Z* axis (right column) are reported for *λ* = 250, 280, and 315 nm, respectively, 280 and 315 nm being the boundaries of the UVB spectral region. Actually, the hot-spots are attenuated and are comparable to the intensity of the incoming radiation when silica absorption is taken into account, as it has been previously experimentally verified (see Figs 3 and 4). Nonetheless, calculation of their position along the optical axis in the absence of absorption allowed isolating the pure geometric contribution of the nanostructured valve to the spatial rearrangement of incoming radiation.

The angle of diffracted light from a circular aperture increases with wavelength^57^. Thus, passing from visible to UV radiation, i.e. lowering the wavelength, the region in which the diffraction contributions of the valve nanopores interfere constructively, giving rise to the hot-spots, moves to higher values of Z. On the right column of Fig. 6 we can observe, indeed, an increase in the distance from the valve of the first hot-spot when the wavelength passes from 315 to 250 nm. In general, in the UV spectral region, radiation is confined along the optical axis at distances greater than those observed for visible radiation and surely greater than the pervalvar axis of the whole frustule (for *C. wailesii*, indeed, pervalvar axis varies from about one half to the whole length of the valve diameter).

**Figure 7:**
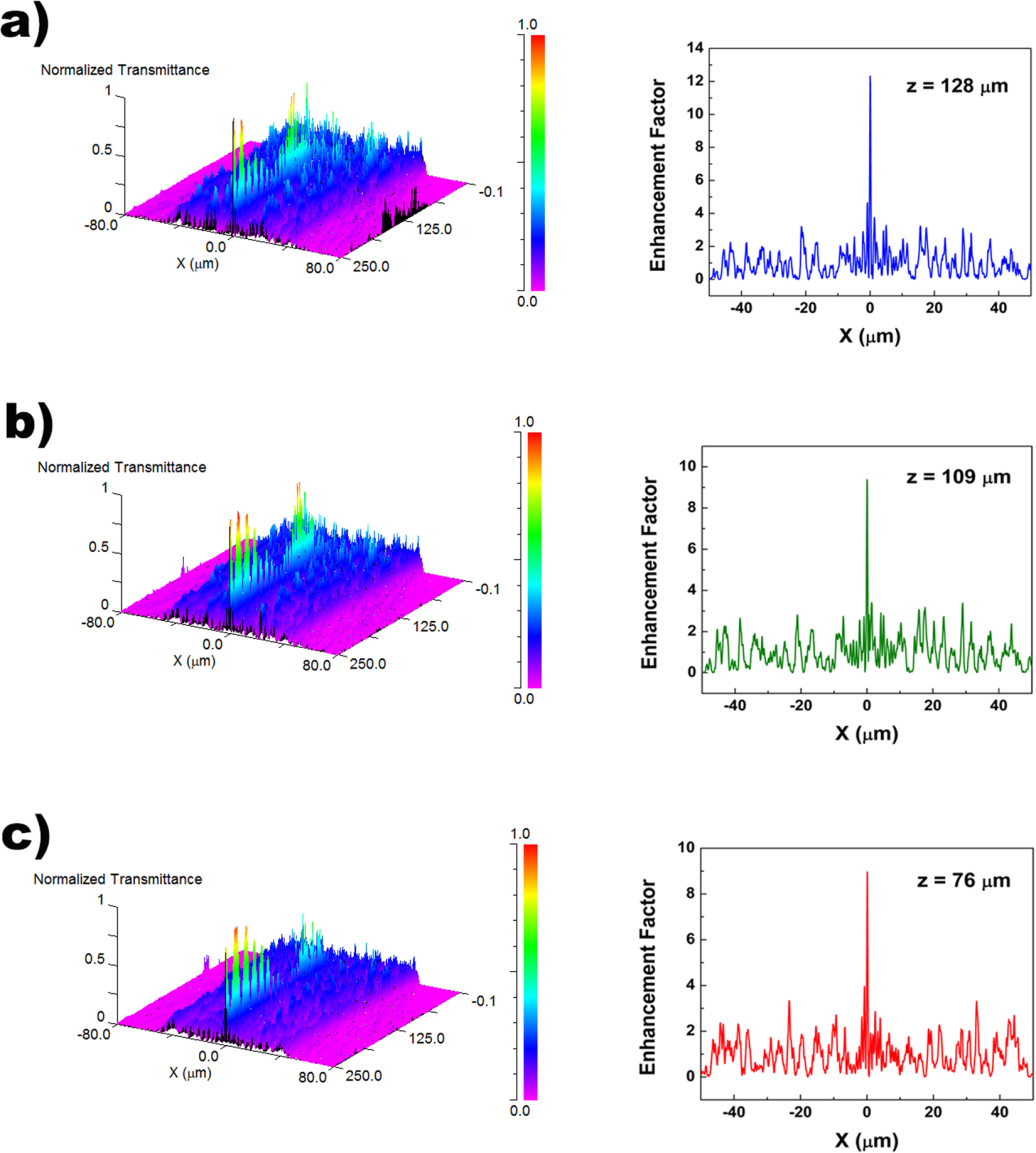
Intensity spatial distribution in the *XZ* plane of visible radiation transmitted by a single valve in air (left column); intensity profile of the first hot-spot with indication of its position along the optical z axis (right column). The simulations have been performed for *λ* = 460 nm (**a**), 532 nm (**b**) and 640 nm (**c**).

In Fig. 7 the simulation results for visible radiation, to which silica is transparent, are reported. In particular, the transmitted intensity distributions and the intensity profiles of the first hot-spot are shown for blue, green and red radiation respectively. Also in this case, increasing the wavelength of the incoming field determined a closer location of the hot-spots with respect to the irradiated valve. Furthermore, since in the considered spectral range there is no significative absorption from silica, the enhancement factors reported in Fig. 7 are real, even though higher than the ones showed in Fig. 4, due to the inevitable approximation in the structure and chemical composition of the model valve used in the simulation. Overall, we can state that the only geometry and refractive index contrast of the valve with respect to the surrounding environment determined the formation of the hot-spots and their spatial location along the optical axis as a function of the incoming wavelength.

**Figure 8:**
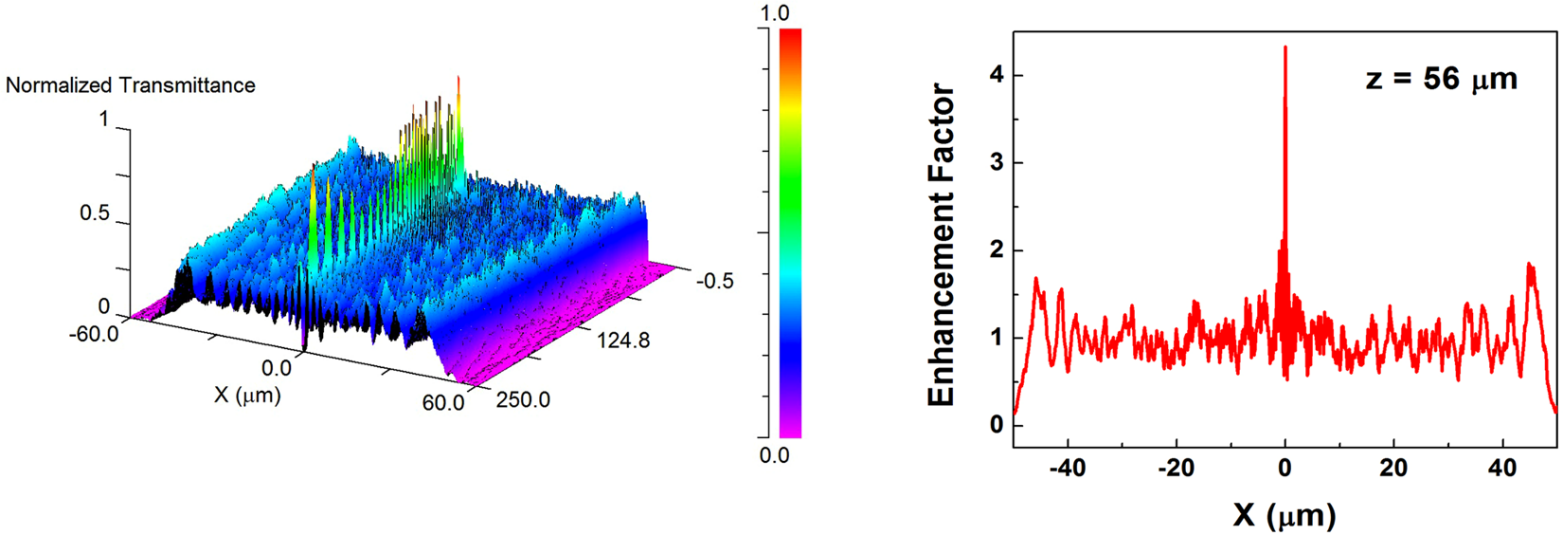
Intensity spatial distribution in the *XZ* plane of red radiation (*λ* = 640 nm) transmitted by a single valve embedded in the cytoplasm (left); intensity profile of the first hot-spot with indication of its position along the optical z axis (right).

In Fig. 8 it is shown what happens when the simulation is performed when considering the valve embedded in cytoplasmatic material (mean refractive index *n*_*cyt*_ = 1.375^58^) and for red radiation: lowering the refractive index contrast between the valve and the surrounding environment causes a further approach of confined light towards the valve, which means that, for living diatoms, most of the light transmitted by the valves is well localized inside the cell. We can look, indeed, at the living cell in Fig. 1b, and compare its thickness (about 80 *μ*m) to the simulated position of the first hot spot (*z* = 56 *μ*m). Furthermore, as specified above, the thickness of the frustule can equal, in many cases, the length of its diameter, increasing the extent of light confinement inside the living cell.

### Wavelength conversion

It is known that several forms of nanostructured silica (e.g. silica nanoparticles, oxidized porous silicon, sol gels, silicon-oxide thin films, silica-based mesoporous materials) present visible photoluminescence after UV excitation^59^. The mechanisms at the basis of this emission of radiation in the visible range cannot be ascribed to band-to-band transitions, due to the wide band gap (about 11 eV) which characterizes amorphous silica^27^. The source of this lies instead in a variety of surface defects, including oxygen defect centers such as non-bridging oxygen hole centers (• O-Si≡) or neutral oxygen vacancy (≡Si-Si≡), in addition to ≡Si-OH (silanol) and ≡Si-H groups^59,60^. However, the most significant contribution to visible emission is due to the recombination of self-trapped excitons (STE) localized by self-induced lattice distortion in presence of strong electron-phonon interactions^61^. In case of silicon dioxide, the electron component of the STE is an oxygen vacancy and the hole is associated with a peroxy linkage (≡Si-O-O-Si≡)^62^.

**Figure 9:**
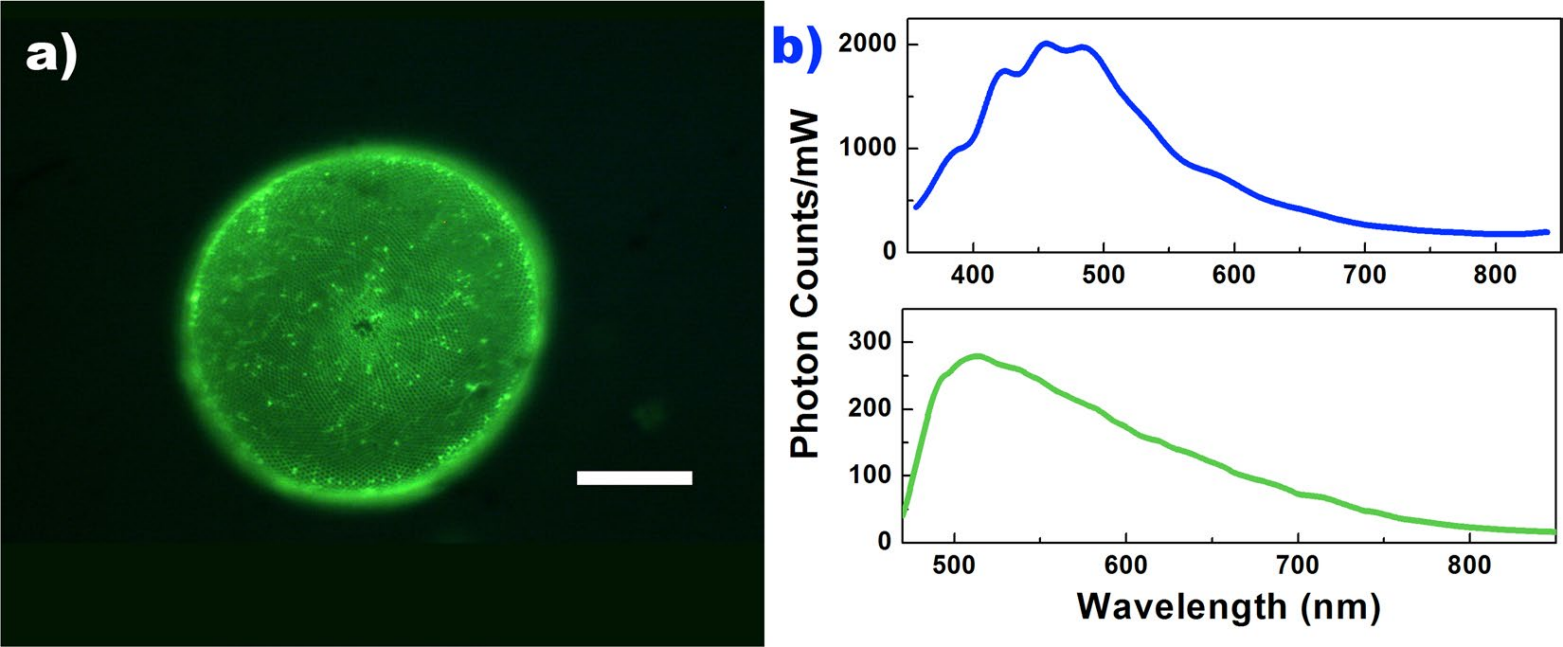
Single valve of *C. wailesii* frustule emitting green radiation after excitation in 450–490 nm spectral range. The bright spots distributed over the valve are ascribable to organic residuals incorporated in the porous silica matrix of the frustule which are scarcely removable even after treatment with strong acid solutions. Scale bar: 50 *μ*m (**a**). Photoluminescence spectra of *C. wailesii* valves after excitation at 325 (top) and 442 (down) nm. Incident power for excitation at 325 nm was 11.5 mW, while at 442 was 60 mW. In both cases, spectra have been acquired with an integration time of 1 second and then corrected respect to incident power (**b**).

As widely reported in literature, nanoporous, hydrogenated silica diatom frustules present visible photoluminescence after excitation in UV-blue range, this property being also exploited in several sensing and biosensing schemes^27,28^. In addition to the surface defects described above and typical of nanostructured silicon dioxide, a further contribution to photoluminescence, in this case, is given by organic residues incorporated in the silica matrix of the frustule^63–65^ (see Fig. 9a).

The visible emission spectra of *C. wailesii* valves after excitation at 325 and 442 nm (Fig. 9b), showed that, in the case of blue emission, several relative maxima are visible, thus suggesting that blue photoluminescence band is a composite of different transitions, which can be observed also in crystalline SiO_2_ luminescence after X-ray irradiation^66^. Furthermore, the spectra reported in Fig. 9 are corrected with respect to incoming irradiation power, thus we could conclude that blue emission after UV excitation is about one order of magnitude more efficient than green emission after blue excitation. This behavior takes a noticeable relevance if we look at the diatoms action spectra, i.e. if we consider the photosynthetic rate per unit of incident irradiance at different wavelengths. The action spectra of diatoms, and, in general, of most microalge, present two main maxima around blue and red spectral regions^48^, in correspondence to absorption peaks of chlorophyll *a*. The characteristics of *C. wailesii* frustule photoluminescence allowed us to state that UV radiation, harmful for living cells mainly through the formation of dimeric photoproducts between adjacent pyrimidines, is efficiently converted in blue radiation, which is in general related to high levels of photosynthetic productivity.

## Conclusions

UVR has been detected in ocean depths down to 60–70 meters^3^. For pure water, indeed, absorption of UVR in the ecologically relevant spectral region (280–300 nm) is negligible with respect to absorption in visible and infrared ranges^48^. As far as sea water is concerned, while salts appear to have no significant effect on absorption in visible/photosynthetic range, nitrates and bromides cause a marked increase in absorption only below 250 nm^48^. Thus the amount of UVR reaching the euphotic zone is quite sufficient to harm microalgae.

Through evolutionary history, most microalgae developed several strategies in order to protect cells from UV irradiation, mainly via excess energy dissipation and synthesis of specific molecules as carotenoids and mycosporine-like amino acids. Nevertheless diatoms, which evolved when atmospheric oxygen and ozone concentrations were low, are characterized by a remarkable tolerance to UV irradiation even in presence of low concentrations of specific UV-absorbing compounds^2–4,8,10^.

We observed that, in the case of *C. wailesii*, a centric diatom, the frustule itself is able to efficiently screen the cell from UV light mainly through three distinct but interplaying mechanisms: absorption by amorphous silica; diffraction-based redistribution of transmitted intensity through the ordered pattern of micro- and nano-pores; and efficient conversion of UV in PAR radiation by photoluminescence. For the first time, to the best of our knowledge, all these aspects of UV/frustule interactions have been explored for *single* valves of *C. wailesii*, while, until now, the UV-shielding effect has been analyzed only for valve monolayers, multilayers or sparse valves^41–43^.

This study contributes to unveil one of the possible evolutionary advantages associated with the micro- and nano-patterned diatom frustules and paves the way for multiple and novel applications in UV filtering, such as degradation prevention in polymers and lacquers and the production of biocompatible sun lotions for skin protection under light exposure.

## Methods

### Frustule preparation

*C. wailesii* strain CCAP 1013/9 has been purchased from the Culture Collection of Algae and Protozoa of the Scottish Association for Marine Science, UK. Cultures were grown in Guillard “f/2” medium with Na_2_SiO_3_ addition at a temperature of 18 °C and illuminated by white light fluorescent tubes (PHILIPS HPL-N150W), with an irradiance of 30–50 *μ*mol photons m^−2^ s^−1^ and 14: 10 LD cycle. Frustules of cultured cells were cleaned by strong acid solutions and heating according to von Stoch’s method^67^, which provided organic matter removal and separation of frustule elements. Single drops of buffer suspension containing the cleaned valves have been deposited and dried onto a quartz slide for light and Raman microscopy and onto a silicon chip for Scanning Electron Microscopy (SEM) characterization.

### Ultrastructural characterization

SEM images of *C. wailesii* have been recorded after valves deposition on flat, doped silicon wafers by drop casting and performed at 5 kV accelerating voltage and 30 *μ*m wide aperture by a Field Emission Scanning Electron Microscope (Carl Zeiss NTS GmbH 1500 Raith FESEM). A secondary electron detector has been used.

### Transmission measurements

Spatial distribution of light intensity transmitted by individual valves in different regions of the optical spectrum has been retrieved by the following experimental set-up. The source of partially coherent radiation was given by a UV-VIS lamp (Hamamatsu, model L10290) provided with optical fiber output; this source includes a deuterium lamp (with emission in the spectral range 200–400 nm) and a tungsten halogen lamp (with emission in 400–1100 nm interval). The two lamps can be used independently or simultaneously, and, in general, the source is provided with a filter holder which can accommodate optical band-pass filters to select limited spectral regions for emission. We used filters at the following spectral windows: 250 nm (Asahi Spectra, 10 nm FWHM), 280–315 nm (Asahi Spectra, UVB filter), 460 nm (Thorlabs, FL460–10, 10 nm FWHM), 532 nm (Thorlabs, FL532-10, 10 nm FWHM), and 640 nm (Thorlabs, FB640-10, 10 nm FWHM). The radiation at the selected spectral window is emitted through a connected fiber (Hamamatsu, A7969 anti-solarization fiber) and collimated by a quartz collimator (Lot Oriel, LLZ010), then spatially filtered by a metallic pinhole (diameter: 200 *μ*m) in order to produce a light beam with comparable dimensions respect to the analyzed valve. The valves were deposited onto a quartz slide, and selected by means of a micrometric xyz translational stage. The transmitted light was collected by a microscope objective (Zeiss, 50X Epiplan, NA 0.7 for visible measurements; Thorlabs, LMU-20 × -UVB with AR coatings in 240–360 nm range for UV measurements) connected with a UV-VIS-NIR sensitive CCD camera (Hamamatsu, C8484-16C, quantum efficiency: 20–40% for 200–280 nm, 20–32% for 280–580 nm and below 20% between 580 and 1100 nm). The acquired images were analyzed and compared with light transmitted by a portion of the quartz slide without any valve. All the optics and the detectors were transparent and/or sensitive in the UV-VIS range. It has to be noted that, in order to avoid to detect visible photoluminescence induced by UV excitation, a further bandpass filter has been inserted in the inlet of the CCD camera (250 ± 10 nm for UVC, 280–315 nm for UVB measurements), in order to cut-off every possible visible contribution.

### Raman spectra acquisition

Raman spectra were acquired using a homebuilt Raman microscope^68^. A laser beam at 532 nm (Opus, Laser Quantum, Maximum Power 2 W) was spatially filtered using a transmitting filter (Maxline, Semrock), expanded to fill the back aperture of a microscope objective and then introduced in an inverted microscope (Olympus I × 51) equipped with a 100× objective lens (Olympus, oil immersion, numerical aperture 1.3) to illuminate a single diatom valve. The lateral and depth resolutions were ~0.48 and ~0.8 *μ*m, respectively. The back-scattered light from the sample was collected from the same objective and filtered by a dichroic beam-splitter (RazorEdge 45° beam-splitter, Semrock), where the radiation at 532 nm was cut. The Raman light was filtered using a laser-blocking filter (RazorEdge 0° notch filter, Semrock) to eliminate the residual Rayleigh scattering and then focused onto the entrance slit of a monochromator (Acton SP2300, Princeton Instruments), set at 100 *μ*m to reject the off-focus light in order to increase the signal-to-noise (SNR) ratio. The monochromator was equipped with a 1800 lines mm^−1^ holographic grating providing an estimated spectral resolution of approximately 1 cm^−1^. The Raman scattered light was finally detected by using a back-illuminated CCD (PIXIS:400BR-eXcelon CCD, Princeton Instruments), thermoelectrically cooled at −70°. A green-filtered illumination led and a video camera system was used to observe the image on the sample during the acquisition. 25 spectra were randomly collected from a single diatom with a step size of 10 *μ*m and an exposure time of 10 s per spectrum. The same analysis has been repeated on three different diatom frustules.

### Numerical simulations

Numerical simulations were based on Beam Propagation Method (BPM) corrected for wide angles and performed by RSoft CAD - Photonics Suite (Synopsis).

Starting from the Helmoltz equation:

1 $${\nabla }^{2}E({\bf{r}})+{k}^{2}({\bf{r}})=0$$

with *E* electric field, *k* = *nk*_0_ wavenumber (with *k*_0_ wavenumber in free space), *n* = *n*(*x*, *y*, *z*) refractive index distribution, we can write the solution as:

2 $$E({\bf{r}})=E(x,y,z)=U(x,y,z){e}^{-i{k}_{r}z}$$

The electric field is thus separated into a slowly varying envelope factor *U*(*x*, *y*, *z*) and a rapid varying phase factor ${e}^{-i{k}_{r}z}$, with *k*_*r*_ = *n*_*r*_*k*_0_ reference wavenumber (expressed in terms of the reference refractive index *n*_*r*_), which takes into account the average phase variation of the field. We are assuming that the considered wave propagates primarily along *z* (*paraxial approximation*), which is not true for a diatom valve where diffraction diverges light; we will see later how to overcome this limit. We will also need to take into account the abrupt change in refractive index when passing from the valve (lying in *xy* plane) to the external environment (air, water or cytoplasm). For now we assume, indeed, not only that the electric field profile along *xy* plane varies slowly, but that the amplitude varies slowly along z axis too. Inserting $U(x,y,z){e}^{-i{k}_{r}z}$ into equation (1) we obtain the following expression:

3 $$\frac{{\partial }^{2}U}{\partial {z}^{2}}+2i{k}_{r}\frac{\partial U}{\partial z}+\frac{{\partial }^{2}U}{\partial {x}^{2}}+\frac{{\partial }^{2}U}{\partial {y}^{2}}+({k}^{2}-{k}_{r}^{2})U=0$$

Making use of the slowly varying envelope approximation:

4 $$|\frac{{\partial }^{2}U}{\partial {z}^{2}}|\ll |2{k}_{r}\frac{\partial U}{\partial z}|$$

we can get the basic BPM equation:

5 $$\frac{\partial U}{\partial z}=\frac{i}{2{k}_{r}}(\frac{{\partial }^{2}U}{\partial {x}^{2}}+\frac{{\partial }^{2}U}{\partial {y}^{2}}+({k}^{2}-{k}_{r}^{2})U)$$

Specifying *U*(*x*, *y*, *z*) at a plane *z* = *z*_0_, we can iterate *U* along the *z*-axis using finite differences for the *x* and *y* derivatives.

The most popular formulation used to derive a wide-angle BPM able to take into account non-paraxiality, is known as the multistep Padé-based technique^69,70^. We can start from equation (3) denoting $\frac{\partial }{\partial z}$ with *D*, and, thus, $\frac{{{\rm{\partial }}}^{2}}{{\rm{\partial }}{z}^{2}}$ with *D*^2^. The equation can be now viewed as a quadratic equation to be solved for the differential operator *D*. This yields to the following formal solution for a first order equation in *z*:

6 $$\frac{\partial U}{\partial z}=i{k}_{r}(\sqrt{1+P}-1)U$$

with:

7 $$P\equiv \frac{1}{{k}_{r}^{2}}(\frac{{\partial }^{2}}{\partial {x}^{2}}+\frac{{\partial }^{2}}{\partial {y}^{2}}+({k}^{2}-{k}_{r}^{2}))$$

Althought restricted to forward propagation of the field, the above equation is still exact in that no paraxiality approximation has been made. In order to evaluate the radical in equation (6), one approach would be to use a Taylor expansion. The first order of the expansion leads to the standard, paraxial BPM, while higher orders lead to more accurate representations. However expansion via Padé approximations^69^ is more accurate than Taylor expansion for the same order of terms. This approach leads to the following wide-angle equation:

8 $$\frac{{\rm{\partial }}U}{{\rm{\partial }}z}=i{k}_{r}\frac{{N}_{m}(P)}{{D}_{n}(P)}U$$

where *N*_*m*_ and *D*_*n*_ are polynomials in the operator *P*, and (*m*, *n*) is the order of approximation. Our numerical capabilities allowed us to make use of the (1, 1) Padé order, corresponding to *N*_*m*_ = *P*/2 and *D*_*n*_ = 1 + *P*/4.

### Photoluminescence spectra acquisition

Steady-state photoluminescence (PL) spectra were excited by a continuous wave He-Cd laser (KIMMON Laser System) at 325 and 442 nm. PL was collected at normal incidence to the surface of samples, consituted by cleaned diatom valves deposited onto a quartz slide, through a fiber, then dispersed by a spectrometer (Princeton Instruments, SpectraPro 300i) and detected using a Peltier cooled charge coupled device (CCD) camera (PIXIS 100 F). Long pass filters with a nominal cut-on wavelength of 350 and 458 nm were used to remove the laser line at monochromator inlet for excitation at 325 and 442 nm, respectively.
